# Protein synthesis and secretion in human mesenchymal cells derived from bone marrow, adipose tissue and Wharton’s jelly

**DOI:** 10.1186/scrt442

**Published:** 2014-04-16

**Authors:** Paola Romina Amable, Marcus Vinicius Telles Teixeira, Rosana Bizon Vieira Carias, José Mauro Granjeiro, Radovan Borojevic

**Affiliations:** 1 Excellion Biomedical Services S.A, Rua Afrânio de Mello Franco 333, 25651000 PetrópolisRio de Janeiro, Brazil; 2 National Institute of Metrology, Quality and Technology (Inmetro), Xerém, RJ, Brazil

## Abstract

**Introduction:**

Different mesenchymal stromal cells (MSC) have been successfully isolated and expanded *in vitro* and nowadays they are tested in clinical trials for a wide variety of diseases. Whether all MSC express the same cell surface markers or have a similar secretion profile is still controversial, making it difficult to decide which stromal cell may be better for a particular application.

**Methods:**

We isolated human mesenchymal stromal cells from bone marrow (BM), adipose tissue (AT) and Wharton’s jelly (WJ) and cultured them in fetal bovine serum supplemented media. We evaluated proliferation, *in vitro* differentiation (osteogenic, adipogenic and chondrogenic potential), expression of cell surface markers and protein secretion using Luminex and ELISA assays.

**Results:**

Cell proliferation was higher for WJ-MSC, followed by AT-MSC. Differences in surface expression markers were observed only for CD54 and CD146. WJ-MSC secreted higher concentrations of chemokines, pro-inflammatory proteins and growth factors. AT-MSC showed a better pro-angiogenic profile and secreted higher amounts of extracellular matrix components and metalloproteinases.

**Conclusions:**

Mesenchymal stromal cells purified from different tissues have different angiogenic, inflammatory and matrix remodeling potential properties. These abilities should be further characterized in order to choose the best protocols for their therapeutic use.

## Introduction

Mesenchymal stromal cells (MSC) are a small population of multipotent progenitor cells present in nearly all human tissues, being mostly found in the perivascular niche [1]. MSC were first isolated from bone marrow [2], but they have been obtained subsequently from a wide variety of fetal and adult tissues: adipose tissue [3], placenta [4], lung [5], umbilical cord [6], synovial membrane [7] and dental pulp [8] among many others.

Regenerative medicine makes use of MSC and of their multipotent properties to promote tissue regeneration. MSC are able to migrate into injured tissues, engraft and differentiate into many cell types, participating thus directly in tissue repair and regeneration [9]. They also secrete paracrine mediators, reducing inflammation and accelerating tissue regeneration by activation of resident stem cells and mobilization of circulating systemic stem cells through chemotactic signaling [10,11]. Clinical trials have already confirmed that use of MSC is safe and effective [12]. Even when MSC express major histocompatibility complex class I, they proved to be safe in allogeneic transplants, also between HLA-incompatible individuals, since they do not elicit alloreactive lymphocyte proliferative responses *in vitro*, they produce locally immunomodulatory proteins and their expression levels of major histocompatibility complex class II are negligible [13,14].

Studies comparing MSC derived from different tissues are limited to *in vitro* and pre-clinical studies. Clinical trials are generally focused on safety and efficiency of a therapy using a specific type of MSC, without demonstrating which MSC is the best for each therapy, or even justifying why a specific cell type was believed to be the best option. Basic MSC comparative studies are required to better understand MSC properties and abilities, indicating the most appropriate MSC type for a particular clinical application.

*In vitro* studies have already shown that MSC from different origins varied when considering differentiation potential: some cells are better for differentiation into osteoblast-like cells [15], while synovium-derived MSC and umbilical cord-derived MSC are better differentiated into chondrocytes than the bone marrow-derived MSC [16,17]. MSC isolated from fetal tissues are superior regarding cardiomyocyte and endothelial cell differentiation when compared to adult tissue-derived MSC [18].

Besides differentiation potential, recent studies have approached other MSC attributes that allowed a deeper understanding of tissue-derived properties. Hsieh and colleagues compared MSC derived from Wharton’s Jelly and bone marrow regarding their ability to regenerate infarcted myocardia; they described secretome differences that make Wharton’s Jelly-derived MSC a more angiogenic, neuroprotective and neurogenic option [19]. Naftali-Shani and coworkers carried out a pre-clinical trial of myocardial infarction in rats comparing the effects of human stromal cells obtained from four locations (epicardial fat, pericardial fat, subcutaneous fat and the right atrium) and they showed that hMSCs from epicardial fat and the right atrium secreted the highest amounts of trophic and inflammatory cytokines *in vitro*, expressed higher amounts of inflammation- and fibrosis-related genes *in vitro* and impaired heart recovery *in vivo*[20]. Research studies like these mentioned here are essential for determining the best tissue-derived mesenchymal stromal cell for a particular regenerative therapy strategy.

In the present study, we compared three different cell types: MSC derived from bone marrow, adipose tissue and Wharton’s Jelly with the purpose to better understand their differences and similarities. We monitored proliferation, differentiation into mesodermal cell types and cell surface marker expression. We also quantified production and secretion of cytokines, chemokines, growth factors and extracellular matrix components into the cell culture supernatants.

## Methods

### Ethics statements

All the experimental procedures were done after Ethics Research Committees approval and all donors signed an informed consent. Adipose tissue-derived MSC (AT-MSC) were obtained from abdominal liposuction during plastic surgery (age: 53.8 ± 5.4 years; sex: 50/50% male/female; CEP No. 55219/12, Research Ethics Committee of Pro-Cardíaco Hospital, Rio de Janeiro, Brazil). Wharton’s Jelly-derived MSC (WJ-MSC) were obtained from umbilical cords of full-term cesarean births (38 to 39 weeks; CEP No. 336/10, Research Ethics Committee of Pro-Cardíaco Hospital, Rio de Janeiro, Brazil). Bone marrow-derived MSC (BM-MSC) were purified from bone marrow harvested from posterior iliac crests during orthopedic surgery (age: 55.5 ± 13.4 years; sex: 50/50% male/female; CEP No. 473/12, Research Ethics Committee of Pro-Cardíaco Hospital, Rio de Janeiro, Brazil).

### Cell isolation and culture

Stromal cells were isolated as previously described [21]. Briefly, nucleated cells were separated from human bone marrow using Ficoll-Paque™ PLUS (GE Healthcare, Uppsala, Uppsala, Sweden, #17-1440-02) by density gradient centrifugation at 700 *g* during a 15-minute period. After washing cells with phosphate-buffered saline (PBS - LGC, Cotia, São Paulo, Brazil, #13-30259-05), they were plated in T25 flasks in alpha-Minimum Essential Medium (α-MEM - LGC, BR30007-05) supplemented with 10% fetal bovine serum (FBS - LGC, #10-BIO-500). Human adipose tissue was washed three times with PBS and was treated with 1.76 mg collagenase type I/gram tissue (Sigma-Aldrich, St. Louis, Missouri, USA, C9891) for 30 minutes at 4°C and 30 minutes at 37°C with agitation. After proteolytic activity inhibition and centrifugation (700 *g*, seven minutes), pelleted cells were plated in T25 flasks. Human umbilical cords were washed with PBS and blood vessels were removed. Wharton’s jelly was cut into small pieces and digested with 0.9 mg collagenase type II/gram tissue (Sigma, C6885) at 37°C for one hour. Washed cells were centrifuged at 700 *g* for seven minutes and plated in T25 flasks. All cells were expanded up to passage number 3 in order to obtain a higher number of cells and were then cryopreserved.

For all the experiments, cells obtained from four different donors in the same passage number were thawed and mixed in order to prepare cell pools, which were immediately plated for proliferation experiments.

### Proliferation curves

Cells of each cell pool were seeded in 24-well plates at a concentration of 6,000 cells/mL in α-MEM supplemented with 10% FBS. Cells grown in different wells were trypsinized and counted in a Neubauer hemocytometer at three different times: 96, 144 and 192 hours.

### Flow cytometry

After cell detachment using a 0.125% trypsin solution, cells were washed with PBS and resuspended in PBS containing 2% FBS. Cell concentration and viability were monitored using Trypan blue in a Neubauer hemocytometer. The following monoclonal antibodies were used as indicated by the manufacturers (BD Pharmingen® (BD, Franklin Lakes, New Jersey, USA)): CD90-PE (BD, #555596), CD73-FITC (BD, #561254), CD105-FITC (BD, #561443), CD45-FITC (BD, #347463), CD14-PE (BD, #555398), CD34-PEcy5 (BD, #561819), CD31-PE (BD, #555446), IgG-FITC (BD, #555786), HLA-DR-FITC (BD, #555558), CD166-PE (BD, #560903), CD44-PE (BD, #555479), CD54-PEcy5 (BD, #555512), CD146-PE (BD, #559263). Isotype controls were used for determining nonspecific binding and defining cut-off values. A minimum of 20,000 events were acquired on a BD FACS Calibur® flow cytometer and results were analyzed using CellQuest™ software.

### Differentiation assays

Pre-cultured cells were seeded into 24-well plates (1 mL/well) at 13,157 cells/cm^2^ (adipogenic differentiation), 5,263 cells/cm^2^ (osteogenic differentiation) or cultured as pellets containing 1 × 10^5^ cells (chondrogenic differentiation). Differentiation medium was replaced twice a week.

#### Adipogenic medium

LG-DMEM supplemented with 10% FBS, 1 μM dexamethasone, 0.5 mM 3-Isobutyl-1-methylxanthine, 10 μM human insulin (Humulin-N®, Eli Lilly and Company, Indianapolis, Indiana, USA), 0.2 mM indomethacin and a penicillin/streptomycin solution at 100 U/mL and 100 μg/mL, respectively.

#### Osteogenic medium

LG-DMEM supplemented with 10% FBS, 10 nM dexamethasone, 10 mM β-glycerophosphate, 50 μM L-ascorbic acid 2-phosphate and penicillin/streptomycin (all reagents obtained from Sigma-Aldrich, St. Louis, Missouri, USA) at 100 U/mL and 100 μg/mL, respectively.

#### Chondrogenic medium

LG-DMEM supplemented with 1% FBS, 50 μg/mL L-ascorbic acid 2-phosphate, 10 ng/mL transforming growth factor-β3, 0.169 UI/mL human insulin and 6.25 μg/mL human transferrin.

After 17 to 21 days, cell cultures were fixed in formalin buffer and washed with PBS. Intracellular accumulated lipids were stained with 0.5% Oil Red O solution. Calcium deposits were stained with 1% Alizarin Red S solution, pH 4.2. Glycosaminoglycans were stained with 1% toluidine blue solution.

### Cytokine, growth factor and extracellular matrix quantification

We quantified cell supernatant concentration of 49 different cytokines, growth factors and extracellular matrix-related proteins: pro-inflammatory cytokines (GM-CSF, IL-1β, IL-6, IL-8, TNF-α, IFN-γ, IL-2, IL-2R, IL-7, IL-12p40/p70, IL-15 and IL-17), anti-inflammatory cytokines (IL-1RA, IL-4, IL-5, IL-10, IL-13 and IFN-α,), chemokines (eotaxin, IP-10, MCP-1, MIG, MIP-1α, MIP-1β and RANTES), angiogenic growth factors (VEGF, VEGF-D, endostatin, aFGF, thrombospondin-2, angiopoietin-1, angiogenin and PLGF), matrix metalloproteinases (MMP-1, -3, -7, -8 and -13) and growth factors (EGF, HGF, bFGF, G-CSF, TGF-β1, TGF-β2, TGF-β3, PDGF-AA, PDGF-AB, PDGF-BB and IGF-1). Commercial Luminex kits were used: Human Cytokine 30-plex Assay (Invitrogen, Carlsbad, California, USA), Fluorokine MAP TGF-β Multiplex Kit (R&D, Minneapolis, Minnesota, USA), Human Angiogenesis Fluorokine Multi Analyte Profiling Kit (R&D, USA), Fluorokine MAP Human MMP kit (R&D, USA) and Milliplex MAP Human IGF-1 Single Plex Kit (Millipore, Billerica, Massachusetts, USA). Only PDGF-AB was quantified using an ELISA kit: Quantikine hPDGF-AB ELISA (R&D, USA). Procedures were performed according to the manufacturers’ instructions.

Extracellular matrix proteins were also quantified in supernatants using commercial ELISA kits and following the manufacturers’ instructions: heparan sulphate (E0623h, EIAab, Wuhan, Hubei, China), aggrecan (E91908Hu, USCN, Wuhan, Hubei, China), decorin (E92127Hu, USCN, USA), elastin (E91337Hu, USCN, USA), laminin (E90082Hu, USCN, USA), perlecan (E82748Hu, USCN, USA), fibronectin (E90037Hu, USCN, USA) and collagens I (E90571Hu, USCN, USA), II (E90572Hu, USCN, USA), III (E90176Hu, USCN, USA), IV (E90180Hu, USCN, USA).

We analyzed cell supernatants obtained from proliferation experiments at Day 8 (end of the exponential growth phase). Quantified supernatants contained FBS, so FBS-supplemented media were also quantified. In order to make results comparable, concentrations were normalized by cell concentration and culture time, therefore expressing them in pg/10^6^ cells/day.

### Statistical analysis

Results were expressed as mean ± standard deviation for at least three replicates. Statistical significance was assessed by one-way non-parametric analysis of variance followed by the Bonferroni test (to compare the three groups). *P*-values <0.05 were considered statistically significant. Statistical analyses were performed using the Prism 5.00 Software (GraphPad Software Inc., San Diego, California, USA).

## Results

### Cell proliferation

**Figure 1:**
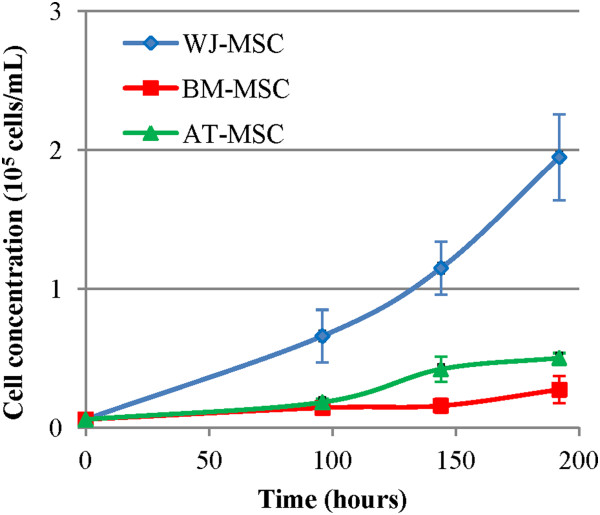
**Proliferation curves of human mesenchymal stromal cells in α-MEM supplemented with 10% fetal bovine serum.** BM-MSC: Bone marrow-derived mesenchymal stromal cells, AT-MSC: adipose tissue-derived mesenchymal stromal cells; WJ-MSC: Wharton’s Jelly-derived mesenchymal stromal cells, α-MEM, alpha-Minimum Essential Medium.

Proliferation curves are shown in Figure 1 and cumulative proliferation times are shown in Table 1. WJ-MSC showed a higher proliferation potential, achieving a final cell concentration four times higher than AT- and BM-MSC after eight days. A slight decrease in cell proliferation was observed for all cell types after nine days in culture, as expected since the number of cell doublings is increasing and cell culture media nutrients are slowly depleted.

**Table 1:** Cumulative population doubling times for mesenchymal stromal cells grown in α-MEM supplemented with 10% FBS

**Time (hours)**	**BM-MSC**	**AT-MSC**	**WJ-MSC**
96	79.5 ± 16.1	59.8 ± 3.3	28.4 ± 3.6
144	79.7 ± 2.1	51.5 ± 3.4	34.0 ± 1.8
192	88.4 ± 8.1	63.5 ± 6.6	38.4 ± 1.7

Cell pools were plated in 24-well plates at a concentration of 6,000 cells/mL and were trypsinized and counted after 96, 144 and 192 hours.

FBS: fetal bovine serum, α-MEM: alpha-Minimum Essential Medium, AT-MSC: adipose tissue-derived mesenchymal stromal cells, BM-MSC: bone marrow-derived mesenchymal stromal cells; WJ-MSC: Wharton’s Jelly-derived mesenchymal stromal cells (WJ-MSC).

It is well known that single “strong” clones can predominate in a long term culture of cell pools. Considering the duplication time of human MSC, an excessive proliferation of a single “strong” clone would take longer than the extension of our experiment, and we therefore assume that the pool is homogeneous during our eight-day proliferation curve.

### Immunophenotyping

Cell cultures in 10% FBS-supplemented medium were analyzed regarding cell surface markers expression by flow cytometry. Results are shown in Table 2. Cell surface marker expressions were similar for all cell types, except CD54 and CD146. CD54 was expressed in 40.0 ± 11.5% BM-MSC, 4.6 ± 3.7% AT-MSC and 97.2 ± 1.7% WJ-MSC. CD146 expression was higher for WJ-MSC (90.0 ± 6.0%) and BM-MSC (81.7 ± 4.4%) and lower in AT-MSC (24.9 ± 3.4%).

**Table 2:** Quantification of cell surface markers by flow cytometry

**Marker**	**BM-MSC**	**AT-MSC**	**WJ-MSC**
**CD73**	99.0 ± 0.9 (65.3 ± 8.6)	92.3 ± 7.0 (153.3 ± 95.4)	96.4 ± 3.3 (283.7 ± 259.6)
**CD90**	98.3 ± 0.8 (125.9 ± 63.6)	97.0 ± 3.1 (203.6 ± 94.7)	99.5 ± 0.4 (295.4 ± 79.2)
**CD105**	94.3 ± 7.3 (66.0 ± 44.3)	94.3 ± 5.7 (142.4 ± 78.8)	87.4 ± 11.7 (151.3 ± 196.0)
**CD45**	0.8 ± 0.9 (3.5 ± 1.6)	0.6 ± 0.7 (11.3 ± 11.0)	1.1 ± 1.1 (7.9 ± 3.8)
**CD14**	1.5 ± 0.8 (2.6 ± 0.8)	0.8 ± 1.0 (13.6 ± 0.1)	0.9 ± 0.7 (8.3 ± 4.4)
**CD34**	0.7 ± 0.6 (2.8 ± 1.6)	0.7 ± 0.9 (2.6 ± 2.1)	2.6 ± 2.7 (8.7 ± 4.7)
**CD31**	0.9 ± 1.1 (10.1 ± 7.9)	0.3 ± 0.5 (3.0 ± 1.0)	1.3 ± 2.0 (5.9 ± 5.5)
**CD44**	99.6 ± 0.2 (233.2 ± 58.1)	99.3 ± 0.1 (1,036.0 ± 1,306.4)	95.6 ± 5.4 (231.7 ± 118.3)
**CD54**	40.0 ± 11.5 (6.0 ± 1.9)	4.6 ± 3.7 (5.7 ± 4.8)	97.2 ± 1.7 (81.3 ± 94.7)
**CD146**	81.7 ± 4.4 (26.5 ± 24.4)	24.9 ± 3.4 (38.9 ± 10.6)	90.9 ± 6.0 (110.4 ± 48.4)
**CD166**	94.7 ± 7.8 (71.2 ± 17.8)	72.4 ± 29.9 (143.2 ± 59.1)	91.0 ± 3.6 (118.9 ± 8.8)
**HLA-DR**	1.8 ± 2.4 (6.0 ± 6.3)	1.1 ± 1.2 (29.1 ± 17.9)	1.1 ± 1.1 (8.0 ± 3.2)
**IgG**	0.7 (8.24)	0.6 ± 0.0 (7.3 ± 5.1)	0.6 (22.8)

Results are expressed in mean percentage ± standard deviation, with MPI values in brackets. AT-MSC: adipose tissue-derived mesenchymal stromal cells, BM-MSC: bone marrow-derived mesenchymal stromal cells, WJ-MSC: Wharton’s Jelly-derived mesenchymal stromal cells.

### Cell differentiation

**Figure 2:**
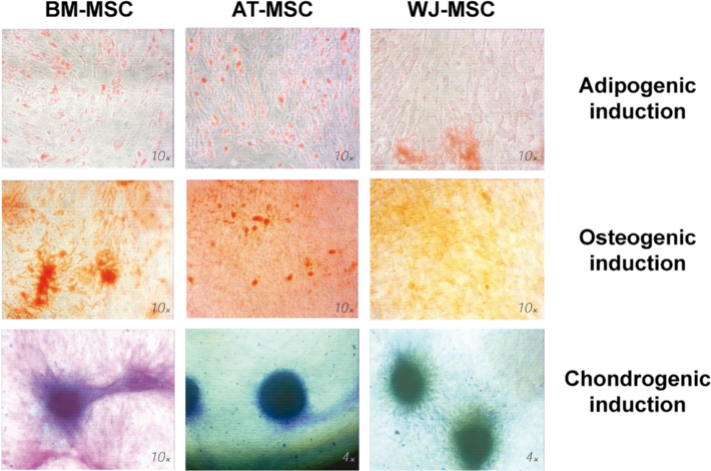
**Cell differentiation assays of human mesenchymal stromal cells to the adipogenic, osteogenic and chondrogenic phenotypes.** After 21 days, intracellular accumulated lipids were stained with Oil Red O, calcium deposits were stained with 1% Alizarin Red S and glycosaminoglycans were stained with toluidine blue. BM-MSC: bone marrow-derived mesenchymal stromal cells, AT-MSC: adipose tissue-derived mesenchymal stromal cells,WJ-MSC: Wharton’s Jelly-derived mesenchymal stromal cells.

All three cell types were differentiated into adipogenic, osteogenic and chondrogenic cell phenotypes (Figure 2). AT- and BM-MSC showed a higher adipogenic potential when compared to WJ-MSC. Regarding osteogenic potential, AT-MSC showed the highest calcium deposition, judging by Alizarin Red S staining. BM-MSC revealed a higher staining for glycosaminglycan with toluidine blue solution but pellet size was smaller than AT- and WJ-MSC.

### Cytokine, growth factor and extracellular matrix quantification

**Figure 3:**
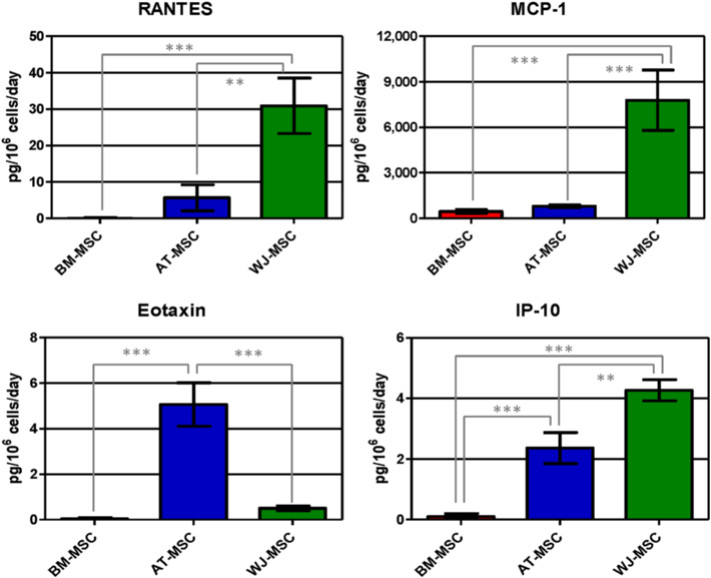
**Concentration of chemokines (RANTES, MCP-1, eotaxin and IP-10) in human mesenchymal stromal cell supernatants.** Values are expressed as average ± standard deviation of three replicates. Statistical significance difference was determined by analysis of variance followed by the Bonferroni *post-hoc* test (n = 3, α = 0.05). BM-MSC: bone marrow-derived mesenchymal stromal cells, AT-MSC adipose tissue-derived mesenchymal stromal cells, WJ-MSC: Wharton’s Jelly-derived mesenchymal stromal cells.

Results from chemokine quantification in cell supernatant of MSC cultures are shown in Figure 3. Among the quantified chemokines, only MIG and MIP-1α were not detected in any supernatant. RANTES was secreted only by AT- and WJ-MSC, with WJ-MSC secretion being 5.4 times higher than AT-MSC. AT- and BM-MSC did not secrete MIP-1β in detectable concentrations; only WJ-MSC secreted this chemokine, but in a low concentration (1.8 ± 1.3 pg/10^6^ cells/day). MCP-1 secretion was similar for BM- and AT-MSC and was significantly higher for WJ-MSC (WJ-MSC secretion was 17.2 and 9.6 times higher than BM- and AT-MSC, respectively). IP-10 secretion was also higher in WJ-MSC, but AT-MSC secreted the highest eotaxin concentration. Comparing all three cell lines, WJ-MSC secreted higher chemokine concentrations. BM-MSC secreted the lowest amount of all chemokines, suggesting a low capacity of cell mobilization into the tissue.

**Figure 4:**
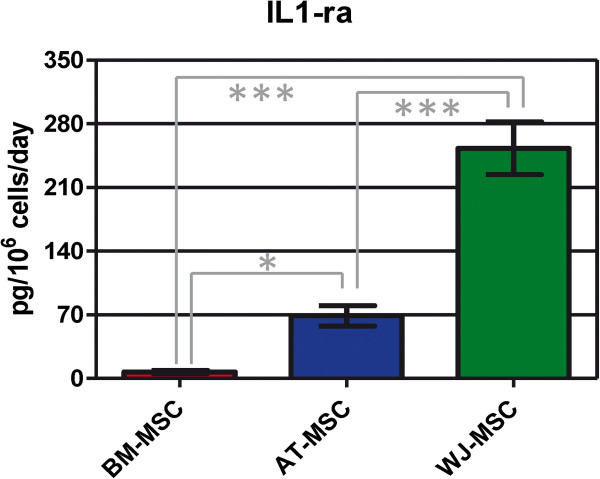
**IL-1ra concentration in human mesenchymal stromal cell supernatants.** Values are expressed as average ± standard deviation of three replicates. Statistical significance difference was determined by analysis of variance followed by the Bonferroni *post-hoc* test (n = 3, α = 0.05). BM-MSC: bone marrow-derived mesenchymal stromal cells, AT-MSC adipose tissue-derived mesenchymal stromal cells, WJ-MSC: Wharton’s Jelly-derived mesenchymal stromal cells.

Considering anti-inflammatory cytokines, only IL-1RA was detected in all three cell types, with WJ-MSC secreting the highest concentration (35.2 and 3.7 times higher than BM- and AT-MSC, respectively; Figure 4). IFN-α was not secreted by AT- and BM-MSC in FBS-supplemented media in detectable concentrations; only WJ-MSC secreted 22.20 ± 0.01 pg IFN-α/10^6^ cells/day. Therefore, WJ-MSC showed the stronger anti-inflammatory profile.

**Figure 5:**
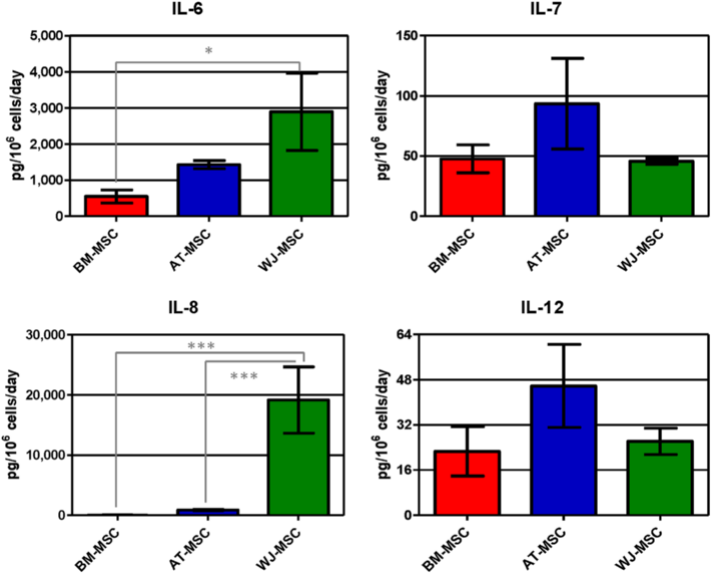
**Concentration of secreted pro-inflamatory cytokines (IL-6, IL-7, IL-8 and IL-12) in mesenchymal stromal cell supernatants.** Values are expressed as average ± standard deviation of three replicates. Statistical significance difference was determined by analysis of variance followed by the Bonferroni *post-hoc* test (n = 3, α = 0.05). BM-MSC: bone marrow-derived mesenchymal stromal cells, AT-MSC adipose tissue-derived mesenchymal stromal cells, WJ-MSC: Wharton’s Jelly-derived mesenchymal stromal cells.

A total of 12 pro-inflammatory cytokines were quantified in all three cell culture supernatants. GM-CSF, TNF-α, IFN-γ, IL-1β, IL-2, IL-2R, IL-15 and IL-17 were not detected in any of them. WJ-MSC secreted the highest concentrations of IL-6 and IL-8 and AT-MSC the highest amounts of IL-7 and IL-12; BM-MSC secreted all four cytokines, but in a lower concentration. These results again confirmed the pro-inflammatory profile of WJ-MSC and the anti-inflammatory behavior of BM-MSC. Results are shown in Figure 5.

**Figure 6:**
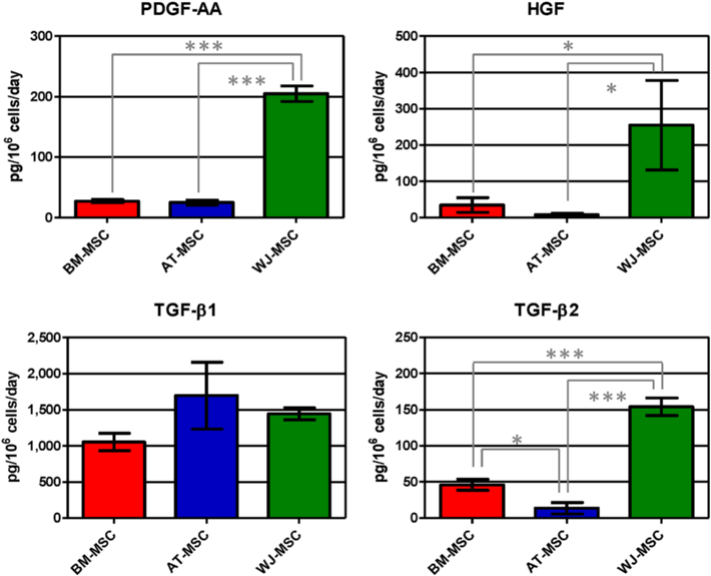
**Growth factor concentration (PDGF-AA, HGF, TGF-β1 and TGF-β2) in human mesenchymal stromal cell supernatants.** Values are expressed as average ± standard deviation of three replicates. Statistical significance difference was determined by analysis of variance followed by the Bonferroni *post-hoc* test (n = 3, α = 0.05). BM-MSC: bone marrow-derived mesenchymal stromal cells, AT-MSC adipose tissue-derived mesenchymal stromal cells, WJ-MSC: Wharton’s Jelly-derived mesenchymal stromal cells.

The growth factors EGF, bFGF, TGF-β3, PDGF-AB and IGF-1 were not detected in any supernatant, meaning that their concentrations were lower than the detection limit of the corresponding assays. PDGF-AA was secreted by all cells under FBS-supplemented media (Figure 6). PDGF-BB was only secreted by BM-MSC, but in a low concentration (1.6 ± 0.8 pg/10^6^ cells/day). Highest concentrations of HGF, TGF-β2 and PDGF-AA were secreted by WJ-MSC. Best producers of TGF-β1 were AT-MSC, but the difference with BM- and WJ-MSC secretion was not significant. G-CSF was secreted only by WJ-MSC at a rate of 28.8 ± 9.1 pg/10^6^ cells/day. Considering the whole growth factor family here quantified, WJ-MSC showed the highest mitogenic profile.

**Figure 7:**
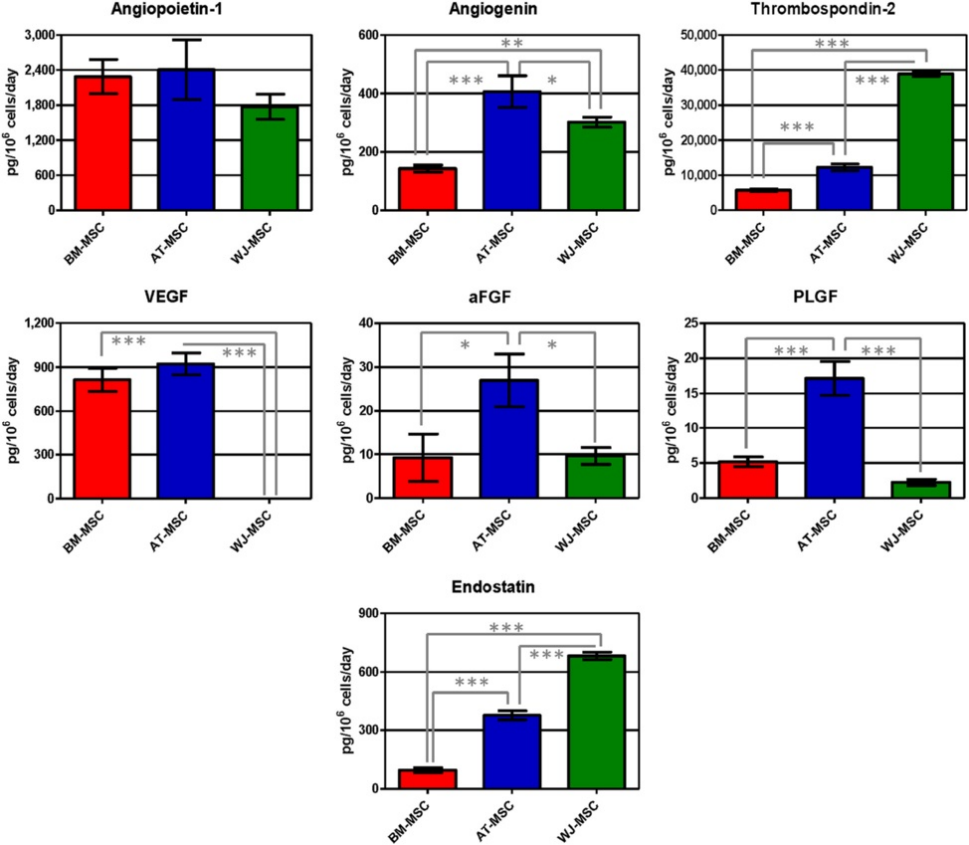
**Concentration of angiogenic factors in human mesenchymal stromal cell supernatants.** In this study we quantified angiopoietin-1, angiogenin, thrombospondin-2, VEGF, aFGF, PLGF, endostatin. Values are expressed as average ± standard deviation of three replicates. Statistical significance difference was determined by analysis of variance followed by the Bonferroni *post-hoc* test (n = 3, α = 0.05). BM-MSC: bone marrow-derived mesenchymal stromal cells, AT-MSC adipose tissue-derived mesenchymal stromal cells, WJ-MSC: Wharton’s Jelly-derived mesenchymal stromal cells.

Results regarding concentration of angiogenic factors in cell supernatants are shown in Figure 7. All the studied angiogenic factors were found in supernatants of the three cell types, except VEGF-D that was only detected in BM-MSC supernatants (23.8 ± 8.4 pg/10^6^ cells/day). WJ-MSC secreted very low concentrations of VEGF, 4,070 and 4,614 times lower than BM- and AT-MSC, respectively. All three cell types showed similar angiopoietin-1 concentrations in their supernatants. AT-MSC secreted the highest amounts of angiogenin, PLGF and aFGF. Thrombospondin-2 secretion by WJ-MSC was 3.2 and 6.8 times higher than AT- and BM-MSC, respectively, and endostatin concentration in WJ-MSC was also higher than AT- and BM-MSC (1.8 and 7.2 times, respectively). Considering these results we can conclude that BM-MSC have a lower angiogenic potential, and that AT- and WJ-MSC are more angiogenic but their effects might be different since they secreted a different panel of angiogenic factors.

**Figure 8:**
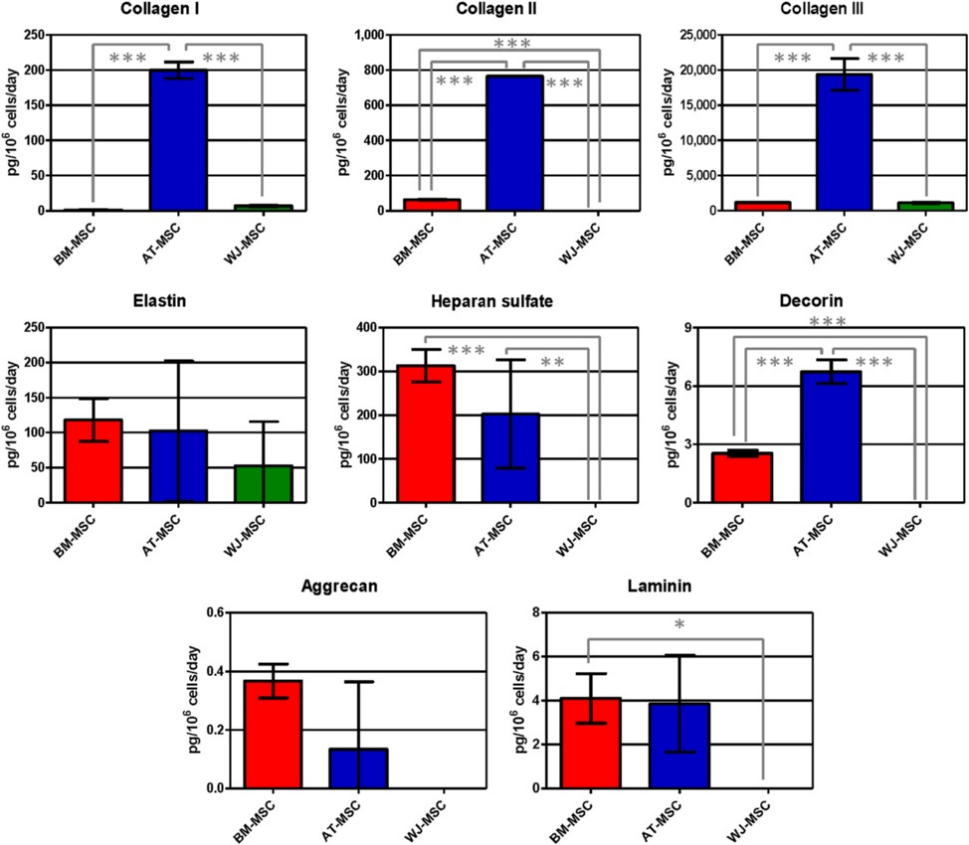
**Secreted amounts of extracellular matrix components in human mesenchymal stromal cell supernatants.** Here we report results obtained for collagen I, II and III, elastin, heparan sulfate, decorin, aggrecan and laminin. Values are expressed as average ± standard deviation of three replicates. Statistical significance difference was determined by analysis of variance followed by the Bonferroni *post-hoc* test (n = 3, α = 0.05). BM-MSC: bone marrow-derived mesenchymal stromal cells, AT-MSC adipose tissue-derived mesenchymal stromal cells, WJ-MSC: Wharton’s Jelly-derived mesenchymal stromal cells.

All the studied extracellular matrix proteins were detected (Figure 8). AT-MSC secreted higher concentrations of collagen I, II and III. AT-MSC were also the only cell types able to secrete collagen IV at detectable concentrations (190.1 ± 48.8 pg/10^6^ cells/day). Fibronectin was not detected in any supernatant. BM-MSC secreted significantly higher amounts of heparan sulfate. Elastin and aggrecan concentrations in BM-MSC supernatants were also higher than in AT- and WJ-MSC, but these differences were not statistically significant. WJ-MSC did not secrete decorin, laminin, fibronectin, heparan sulfate, collagen II and IV and aggrecan in FBS-supplemented medium. Thus, AT-MSC are an excellent collagen-secreting cell type and WJ-MSC secrete a limited amount of extracellular matrix components.

**Figure 9:**
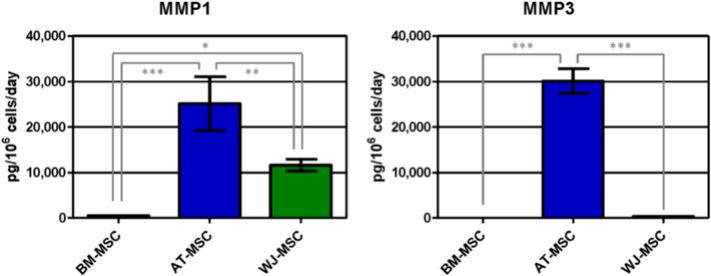
**Concentration of secreted matrix metalloproteinases (MMP1 and MMP3) in human mesenchymal stromal cell supernatants.** Values are expressed as average ± standard deviation of three replicates. Statistical significance difference was determined by analysis of variance followed by the Bonferroni *post-hoc* test (n = 3, α = 0.05). BM-MSC: bone marrow-derived mesenchymal stromal cells, AT-MSC adipose tissue-derived mesenchymal stromal cells, WJ-MSC: Wharton’s Jelly-derived mesenchymal stromal cells.

Metalloproteinase concentrations are shown in Figure 9. AT-MSC secreted the highest concentrations of MMP1 (collagenase 1) and MMP3 (stromelysin 1). None of the three cell types secreted MMP7 (matrilysin). MMP8 (collagenase 2) was secreted by BM-MSC only (50.3 ± 16.8 pg/10^6^ cells/day). MMP13 (collagenase 3) secretion was only quantified in AT-MSC supernatants (25.6 ± 0.1 pg/10^6^ cells/day).

Results from all proteins quantified are also shown in Additional file 1: Table S1.

## Discussion

The present study was done to compare biological parameters of the three MSC isolated from the most frequently used tissue sources: bone marrow, adipose tissue and Wharton’s Jelly of the umbilical cord, under the basal conditions of *in vitro* cell culture using synthetic media and fetal bovine serum. This first study is being pursued by *in vitro* analyses under conditions more closely representative of the *in vivo* tissue environments found in cell therapies using MSC. We describe the characterization of a representative range of secreted proteins by three mesenchymal stromal cells, isolated from different human tissues. The proposal is the selection of the most appropriate type of MSC for a specific therapy, since key characteristics are needed for the cells to effectively participate in tissue regeneration for a particular disease. For example, for cartilage-regenerating therapies, a cell secreting high amounts of cartilage-specific components of the extracellular matrix would be desired; ischemic diseases should be ideally treated with a cell showing anti-inflammatory and pro-angiogenic potential, in order to control the inflammatory environment and to promote the re-vascularization of the affected area. Our results confirmed that mesenchymal cells derived from different origins have different properties in terms of pro-regenerative, pro-angiogenic or anti-inflammatory activities. Analyses of the behavior of the three different MSC studied here may indicate the good choice of the type of the MSC to be used in each therapy.

Nowadays, MSC are used in regenerative medicine in two contexts: autologous and allogeneic. In the former one, integration of MSC into the regenerating tissues is sought, and the capacity of proliferation and production of extracellular matrix are relevant. In the latter one, a long term integration of MSC is not expected, even if MSC express only low levels of the histocompatibility markers. Their anti-inflammatory activity and their capacity to activate resident stem cells and mobilize the circulating ones into the regenerating tissues are thus relevant.

We evaluated MSC proliferation and we confirmed that WJ-MSC have a higher proliferation rate, results that were already reported by other authors [17,22,23]. This is expected since WJ-MSC are of fetal origin, and the cell proliferation decreases sharply with maturation of the donor. With a rare exception of the cryopreserved cells of the Wharton’s Jelly for future use in which autologous cells may be available, their use is at present essentially dedicated to the allogeneic use. Nevertheless, their availability at the perinatal period may be considered in therapy of preterm birth, repair of inborn defects and in perinatal accidents, and, in view of their high protrophic and angiogenic capacity described in the present study, they may offer promising solutions [24,25].

The relatively high expression of CD54 and CD146 in WJ-MSC may be consistent with the proposal of their high potential in regenerative medicine. The CD54 (ICAM-1) is an adhesion molecule that upon stimulation with pro-inflammatory mediators promotes mobilization and transendothelial migration of circulating cells into injured tissues [26]. CD146 (MCAM, MUC18) is a cell adhesion molecule, whose high expression on MSC is associated with greater differentiation potential [27].

When considering an inflammatory profile, WJ-MSC secreted the highest concentrations of RANTES, MCP-1 and IP-10, chemokines able to attract a wide range of inflammatory cells (monocytes, macrophages, dendritic cells, T lymphocytes). IL-6 was secreted at higher concentrations by WJ-MSC, but all three cells secreted high IL-6 concentrations: while plasmatic concentration in healthy patients varied from 0.02 up to 10.1 pg/mL [28], IL-6 concentration in cell supernatants was much higher: 697.6 ± 232.8 pg/mL for BM-MSC, 620.7 ± 49.4 for AT-MSC and 4,001.0 ± 1,484.1 for WJ-MSC. IL-6 was recently associated with MSC pluripotency and immunoprivilege: BM-MSC differentiation induced loss of immune privilege and down-regulation of IL-6 secretion [29,30]. Nasir and colleagues demonstrated that a combination of BM-MSC and IL-6 is much more effective in attenuating liver fibrosis than BM-MSC alone [31], and this result makes us suggest that maybe WJ-MSC alone would be a more appropriate candidate for this therapy, since these cells secrete higher IL-6 concentrations.

IL1-RA is an antagonist of the IL-1 receptor and belongs to the IL-1 cytokine family, a group of at least 11 inflammatory mediators. IL1-RA has already been described as a therapeutic candidate for diabetes mellitus II treatment, because of its protective effects: IL1-RA prevented pancreatic mononuclear cell infiltration, islet destruction and hyperglycemia in a model of induced diabetes mellitus [32]. In pre-clinical studies in mice, Ortiz and coworkers demonstrated that BM-MSC had an anti-inflammatory and anti-fibrotic effect and that this effect was mainly mediated by IL1-RA; they also demonstrated that BM-MSC were more effective than recombinant IL1-RA [33]. Therefore, this potential of WJ-MSC, mediated by IL1-RA secretion, should also be exploited.

IL-8 was secreted in very different concentrations among different cells: while BM-MSC secreted 47.8 ± 11.7 pg/10^6^ cells/day, and WJ-MSC secreted 19,151.3 ± 5,512.2 pg/10^6^ cells/day, 400 times higher than BM-MSC production. A few authors reported increased IL-8 from MSC when cells were stimulated with lipopolysaccharides (LPS) [34], but identification of whether IL-8 secretion would be helpful in a determined MSC application needs to be studied.

Regarding angiogenic factors, only BM-MSC secreted detectable amounts of VEGF-D. VEGF-D was defined as the strongest angiogenic and lymphangiogenic VEGF isoform [35] and it has been tested in phase I clinical trials for myocardial infarction in a gene therapy approach (ClinicalTrials.gov; Identifier: NCT01002430).

WJ-MSC produced almost undetectable amounts of VEGF; on the other hand, AT- and BM-MSC were the highest VEGF producers. Research articles like those published by Deuse’s and Augustin’s groups showed that MSC genetically modified to overexpress VEGF are more appropriate than MSC alone for treating acute myocardial infarction, as VEGF extended MSC survival and protected them against apoptosis and improved heart function recovery [36,37]. This information suggests that BM- and AT-MSC would be better candidates for infarction therapy than WJ-MSC.

Thrombospondin-2 was another angiogenic factor that was found in high concentrations in all cell culture supernatants and WJ-MSC secreted the highest amounts. Jeong and colleagues have shown that WJ-MSC exerted a regenerative effect on cartilage and they suggested that this effect was mediated by thrombospondin-2, since this molecule alone could exert a similar effect and thrombospondin-2 knock-down in WJ-MSC using siRNA abolished its chondro-regenerative potential [38]. Considering that AT-MSC also secreted high concentrations of thrombospondin-2, they would be a good potential candidate for cartilage regeneration, especially taking into account autologous adipose tissue availability in cases when the patients did not cryopreserve their own umbilical cord cells.

Angiopoietin-1 is an important pro-angiogenic factor involved in vascular maturation and neovasculogenesis. Similar to what was described for VEGF, genetically modified MSC expressing angiopoietin-1 were more effective in regenerating myocardial tissue after infarction than MSC alone [39]. Liu and coworkers also demonstrated that MSC expressed functional receptors for angiopoietin-1 and that angiopoietin-1 protected MSC against apoptosis induced by hypoxia and serum deprivation [40]. Angiopoietin-1 secretion was not statistically different for all three MSC, but the secretion rate of WJ-MSC was lower than those of AT- and BM-MSC.

PLGF displays a high homology with VEGF, binds to the VEGF receptor and is able to augment the action of VEGF both *in vivo* and *in vitro*. Liu and colleagues showed that PLGF exerted a neuroprotective and angiogenic effect in cerebral ischemia: BM-MSC injected intravenously reduced lesion volume and induced angiogenesis while improving functional recovery, but the effect was greater when BM-MSC were genetically modified to over-express PLGF [41]. Considering our results, AT-MSC secreted higher concentrations of PLGF: 3.3 and 7.8 times higher than BM- and WJ-MSC. This origin-related differential of AT-MSC would make them more appropriate for tissue regeneration after cerebral ischemia.

When analyzing growth factor secretion, WJ-MSC secreted higher amounts of TGF-β2, HGF and PDGF-AA and was the only cell type secreting G-CSF in detectable levels. HGF promotes migration, proliferation and survival of a wide range of cell types and was shown to induce expression of cardiac-specific markers in MSC [42]. G-CSF is a potent chemotactic factor for MSC and has been successfully used in different applications, such as acute myocardial infarction [43] and injured brain [44].

Regarding extracellular matrix components, AT-MSC secreted the highest amounts of collagen I, II, III and IV and WJ-MSC did not secrete any detectable amounts of collagen II and IV. WJ-MSC did not produce fibronectin, heparan sulphate, decorin, laminin and aggrecan. Collagen II is the most abundant protein in articular and hyaline cartilage; therefore, WJ-MSC would not be appropriate for cartilage regeneration since it would generate a fibrous tissue due to its higher collagen III secretion. Collagen I and elastin are the main components of tendons, making AT-MSC the most appropriate cell type for tendon regeneration. MSC are also key players in skin regeneration and aging prevention: they are attracted by PDGF-BB secreted by endothelial cells and they increase collagen IV secretion, a component of basement membranes [45]. AT-MSC were the only cell type that secreted PDGF-BB *in vitro* and they produced the highest amounts of collagen IV, suggesting they are the most appropriate cell type for skin regeneration, since neither BM- nor WJ-MSC secreted PDGF-BB and collagen IV.

MMP1, MMP3 and MMP13 are important enzymes involved in the degradation of bone, cartilage and tendon. Serum and synovial levels of MMP1, MMP3 and MMP13 are increased in rheumatoid arthritis and osteoarthritis [46,47]. Analyzing our results, AT-MSC produced the highest concentrations of MMP1 and MMP3, this being a negative point for using AT-MSC in bone, cartilage and tendon applications. AT- and WJ-MSC secreted high amounts of MMP1 and MMP3, but low levels or no MMP13. A good candidate for bone, cartilage and tendon regeneration, when considering matrix metalloproteinases, would be BM-MSC, which secreted the lowest concentrations of MMP1 and MMP3 and no MMP13.

In summary, WJ-MSC showed a higher pro-inflammatory and mitogenic profile, while AT-MSC secreted higher concentrations of pro-angiogenic proteins, extracellular matrix components and matrix metalloproteinases. Acquiring a deeper molecular knowledge about MSC from different origins will help us make a more rational and effective selection of the MSC for different applications in regenerative medicine.

## Conclusions

Our data showed that MSC from different tissues have different properties *in vitro* and this can be the reason for their different behavior in different clinical applications. In conclusion, cell type for regenerative therapies should be carefully chosen. The present results need to be compared with *in vivo* pre-clinical studies in order to suggest the best protocols for mesenchymal stromal cell use in each clinical therapy.

## Abbreviations

aFGF: Acidic fibroblast growth factor; AT-MSC: Adipose tissue-derived MSC; bFGF: Basic fibroblast growth factor; BM-MSC: Bone marrow-derived MSC; DMEM: Dulbecco’s modified Eagle media; EGF: Endothelial growth factor; FBS: Fetal bovine serum; FCS: Forward scatter; G-CSF: Granulocyte colony-stimulating factor; GM-CSF: Granulocyte macrophage colony-stimulating factor; HGF: Hepatocyte growth factor; IFN: Interferon; IGF-1: Insulin growth factor 1; IL: Interleukin; IP-10: Interferon gamma-induced protein 10; LG-DMEM: Low-glucose DMEM; MCP-1: Monocyte chemoattractant protein-1; MEM: Minimum essential media; MIG: Monokine induced by IFN-gamma; MIP-1: Macrophage inflammatory protein 1; MMP: Matrix metalloproteinase; MSC: Mesenchymal stromal cells; PBS: Phosphate-buffered saline; PDGF: Platelet-derived growth factor; PLGF: Placental growth factor; RANTES: Regulated on activation, normal T cell expressed and secreted; SSC: Side scatter; TGF: Transforming growth factor; TNF-α: Tumor necrosis factor alpha; VEGF: Vascular endothelial growth factor; WJ-MSC: Wharton’s jelly-derived MSC.

## Competing interests

PRA, MVTT, RBVC and RB are employees at Excellion Biomedical Services S.A. Results reported here have no connections or influence on the company’s products. The authors declared that no competing financial and/or private interests exist.

## Authors’ contributions

PRA, MVTT, RBVC, RB and JMG participated in the conception and design of the study. PRA, RBVC and MVTT performed the experiments, collected and analyzed the data. PRA performed the statistical analysis and wrote the manuscript. MTVV and RBVC revised the manuscript. JMG and RB revised and approved the final manuscript. All authors read and approved the final manuscript.

## Supplementary Material

Additional file 1: Table S1 Protein concentration in human mesenchymal stromal cell supernatants. Results are expressed in expressed in mean pg/10^6^ cells/day ± standard deviation of three replicates. BM-MSC: bone marrow-derived mesenchymal stromal cells, AT-MSC adipose tissue-derived mesenchymal stromal cells, WJ-MSC: Wharton’s Jelly-derived mesenchymal stromal cells. Click here for file
