# Supplementary material for: Protein synthesis and secretion in human mesenchymal cells derived from bone marrow, adipose tissue and Wharton’s jelly
Source: Stem Cell Res Ther. 2014 Apr 16;5(2):53. doi: 10.1186/scrt442 (PMC4055160; doi:10.1186/scrt442)
Supplement: Additional file 1: Table S1 — Protein concentration in human mesenchymal stromal cell supernatants. Results are expressed in expressed in mean pg/106 cells/day ± standard deviation of three replicates. BM-MSC: bone marrow-derived mesenchymal stromal cells, AT-MSC adipose tissue-derived mesenchymal stromal cells, WJ-MSC: Wharton’s Jelly-derived mesenchymal stromal cells. [file scrt442-S1.docx]

| **pg/10^6^ cells/day** | **BM-MSC** | **AT-MSC** | **WJ-MSC** |
| --- | --- | --- | --- |
| **Chemokines** | | | |
| **Eotaxin** | 0.04 ± 0.01 | 5.1 ± 0.9 | 0.5 ± 0.1 |
| **IP-10** | 0.1 ± 0.1 | 2.4 ± 0.5 | 4.3 ± 0.4 |
| **MIP-1b** | nd | nd | 1.8 ± 1.3 |
| **MCP-1** | 453.9 ± 122.0 | 808.7 ± 86.4 | 7787.2 ± 1996.1 |
| **RANTES** | nd | 5.7 ± 3.6 | 30.9 ± 7.7 |
| **Pro-inflammatory cytokines** | | | |
| **IL-6** | 546.0 ± 182.2 | 1428.7 ± 113.7 | 2896.2 ± 1073.4 |
| **IL-7** | 47.6 ± 11.7 | 93.5 ± 37.6 | 45.7 ± 2.5 |
| **IL-8** | 47.8 ± 18.3 | 887.5 ± 111.6 | 19151.3 ± 5512.2 |
| **IL-12** | 22.6 ± 8.7 | 45.8 ± 14.7 | 26.2 ± 4.7 |
| **Anti-inflammatory cytokines** | | | |
| **IFN-a** | nd | nd | 22.20 ± 0.01 |
| **IL-1ra** | 7.1 ± 1.8 | 68.8 ± 11.2 | 253.1 ± 29.0 |
| **Angiogenic factors** | | | |
| **Angiogenin** | 142.5 ± 12.4 | 405.9 ± 54.3 | 301.9 ± 17.1 |
| **Thrombospondin-2** | 5749.8 ± 349.2 | 12271.3 ± 968.4 | 38947.6 ± 668.8 |
| **PIGF** | 5.1 ± 0.7 | 17.1 ± 2.5 | 2.2 ± 0.5 |
| **aFGF** | 9.2 ± 5.4 | 27.0 ± 6.0 | 9.6 ± 1.9 |
| **VEGF-D** | 23.8 ± 8.4 | nd | nd |
| **Endostatin** | 94.9 ± 13.1 | 377.2 ± 23.3 | 682.3 ± 19.2 |
| **Angiopoietin-1** | 2288.0 ± 292.2 | 2407.1 ± 511.7 | 1775.0 ± 215.0 |
| **VEGF** | 814.0 ± 79.1 | 922.7 ± 74.0 | 0.2 ± 0.3 |
| **Growth factors** | | | |
| **TGF-b1** | 1054.0 ± 120.0 | 1694.0 ± 461.6 | 1443.1 ± 82.3 |
| **TGF-b2** | 46.0 ± 7.7 | 13.7 ± 7.9 | 154.0 ± 11.9 |
| **HGF** | 34.8 ± 20.5 | 8.0 ± 3.8 | 254.6 ± 123.8 |
| **G-CSF** | nd | nd | 28.8 ± 9.1 |
| **PDGF-AA** | 27.3 ± 2.5 | 25.0 ± 3.8 | 205.1 ± 12.9 |
| **PDGF-BB** | 1.6 ± 0.8 | nd | nd |
| **Extracellular matrix proteins** | | | |
| **Collagen I** | 1.1 ± 0.2 | 200.0 ± 11.6 | 6.9 ± 0.8 |
| **Collagen II** | 62.2 ± 2.8 | 523.8 ± 418.2 | nd |
| **Collagen III** | 1185.1 ± 51.1 | 19380.8 ± 2238.1 | 1134.6 ± 76.3 |
| **Collagen IV** | nd | 190.1 ± 48.8 | nd |
| **Elastin** | 118.2 ± 30.7 | 69.9 ± 90.2 | 23.1 ± 67.9 |
| **Heparan sulfate** | 312.8 ± 37.1 | 202.7 ± 123.7 | nd |
| **Decorin** | 2.5 ± 0.1 | 6.7 ± 0.6 | nd |
| **Laminin** | 4.1 ± 1.1 | 1.2 ± 4.8 | nd |
| **Aggrecan** | 0.4 ± 0.1 | 0.1 ± 0.4 | nd |
| **Metalloproteinases** | | | |
| **MMP1** | 487.5 ± 11.2 | 25104.6 ± 5934.5 | 11625.4 ± 1293.3 |
| **MMP3** | 24.3 ± 2.4 | 30100.6 ± 2711.8 | 337.1 ± 24.9 |
| **MMP8** | 50.3 ± 16.8 | nd | nd |
| **MMP13** | nd | 25.6 ± 0.1 | nd |
